# Cost-effectiveness and value of information analysis of a low-friction environment following skin graft in patients with burn injury

**DOI:** 10.1186/s40814-019-0543-1

**Published:** 2020-01-31

**Authors:** Rebecca Kandiyali, Howard Thom, Amber E. Young, Rosemary Greenwood, Nicky J. Welton

**Affiliations:** 1 Population Health Sciences, Bristol Medical School, University of Bristol, Bristol, UK; 2 University Hospitals Bristol NHS Foundation Trust, Bristol, UK

**Keywords:** Cost-effectiveness analysis, Proof of concept study, Randomised controlled trial, Value of information analysis

## Abstract

**Background:**

Patients with burn injuries may receive a skin graft to achieve healing in a timely manner. However, in around 7% of cases, the skin graft is lost (fails to attach to the wound site) and a re-grafting procedure is necessary. It has been hypothesised that low-friction (smooth, more slippery) bedding may reduce the risk of skin-graft loss. A before and after feasibility study comparing low-friction with standard bedding in skin-grafted patients was conducted in order to collect proof of concept data. The resulting relative risk on the primary outcome (number of patients with skin graft failure) for the non-randomised study provided no evidence of effect but had a large standard error. The aim of this study is to see if an appropriately powered randomised control trial would be worthwhile.

**Methods:**

A probabilistic decision-analytic model was constructed to compare low-friction bedding to standard care in a population of burn patients who have undergone skin grafting. Results from the before and after study were used as model inputs. The sensitivity of results to bias in the relative risk of graft loss was conducted. Low-friction bedding is considered optimal if expected incremental net benefit (INB) is positive. Uncertainty is assessed using cost-effectiveness acceptability curves. Expected Value of Perfect Partial Information (EVPPI) provides an upper bound for the potential net health benefits of new research for given model input.

**Results:**

At a willingness to pay threshold of £20,000 per QALY, INB = £151 (95% Credible Interval (CrI) −142 to 814), marginally favouring low-friction bedding but with high uncertainty (probability of being cost-effective 70.5%). Expected value of perfect information (EVPI) per patient was £20.29, which results in a population EVPI of £174,765 over a 10-year lifetime for the technology (based on 1000 patients per year who would benefit from the intervention). The parameter contributing most to the uncertainty was the inpatient care cost, i.e. information that could be obtained from the audit of practice and without an expensive trial. These findings were robust to a wide-range of assumptions about the potential bias due to the observational nature of the comparative evidence.

**Conclusions:**

Our study results suggest that an RCT (randomised controlled trial) is unlikely to be worthwhile, but there may be value in a study to estimate the re-graft rates and associated costs in this population.

## Background

A burn injury to the skin or other tissue occurs when cells are destroyed by hot liquids (scalds), hot solids, flames, electricity, chemicals or cold [1]. Around 130,000 patients with burns present to English and Welsh hospitals annually; 45,000 are severe enough to require hospital follow-up [1]. Of these, 11,500 patients require admission of which 50% are children [2]. Globally, 11 million people are estimated to suffer burns significant enough to warrant medical attention [3]. Early wound closure after an injury is the aim of all modern burn care pathways resulting in improvement in survival at a substantially lower cost, better cosmetic outcomes and shorter lengths of hospital stay [4]. Skin grafts are used to treat burns to achieve healing in full-thickness burns (where the three layers of skin known as the epidermis, dermis and subcutis are damaged) or for the best cosmetic outcome in partial-thickness burns (where the top two layers, i.e. the epidermis and dermis are damaged) failing to heal within 3 weeks [5]. There are roughly 1000 skin grafts undertaken to achieve healing annually at burns services nationally; 75% in adults and 25% in children [2]. National Burns Injury database (iBID) data suggest that 20 to 30% of patients will require further grafting procedures. Some of this graft failure will be due to infection and some due to friction when the graft rubs against other materials (graft loss because of friction between bedsheets and another material, such as a bedsheet). It is difficult to determine how many of graft failures are due to friction alone. Wounds that fail to heal will cause considerable distress to patients, impacting negatively on physical, social, emotional and economic aspects of their life [6]. Graft loss will result in delayed wound healing, increased hospital stay, repeat surgery, further donor sites, increased pain and the potential for infection and increased scar formation; impacting negatively on UK National Health Service (NHS) costs.

Low-friction bedding has been shown to be a clinical and cost-effective option in the prevention of skin breakdown in a non-burns population at-risk of skin breakdown [7]. Therefore, a hypothesis is that low-friction bedding may reduce the risk of skin-graft loss in patients with burn injury; however, the data is presently limited to non-randomised evidence from a before and after study design [8]. In brief, the Skin grafting Low friKtion Environment (SILKIE) study was a non-randomised two-centre study of the feasibility of delivering the low-friction environment with proof of concept through the comparison of retrospective with prospective data collection over 12 months. Given the low level of evidence, there remains uncertainty whether low-friction bedding prevents skin graft losses. Although low-friction bedding costs more than standard bedding, if it reduces graft loss, then this is expected to make a saving to the NHS, since re-grafting is expensive (due to the cost of the re-graft procedure and associated in-patient stay). We are therefore uncertain about both effectiveness and cost-effectiveness. This raises the question as to whether a RCT would be a worthwhile investment.

The aim of this paper is to build an economic model to assess the cost-effectiveness of low-friction bedding and use this to (i) assess uncertainty in optimal bedding given current evidence and (ii) to quantify the value of conducting a new RCT to reduce uncertainty as to the most cost-effective bedding.

The paper is organised as follows. We begin by describing the decision question and model structure. We then describe the evidence sources used to populate the model. We then present results from the cost-effectiveness analysis and value of information analysis. We finish with a discussion of the implication of the results for future research priorities.

## Methods

The economic model was designed to assess cost-effectiveness for the following population and interventions.

### Population

Patients (adults and children) with burns who have undergone a skin graft.

### Intervention and comparator

The intervention was the use of low-friction bedding in patients who are recovering from skin graft surgery. The comparator was standard bedding.

### Outcomes

We calculated the Expected net benefit (ENB) and the probability of being cost-effective. ENB puts costs and QALYs onto a monetary scale using the formula: Expected Net Benefit =Expected (QALYs) * lambda +Expected (costs), where lambda represents the ceiling ratio that society is willing to pay for a gain in QALY—typically £20,000 in recommendations presided over by NICE [9]. The Expected Incremental Net Benefit is the difference between ENB on low-friction and standard bedding, with positive values indicating that low-friction is optimal. Uncertainty in the optimal bedding is assessed by reporting credible intervals around the ENB, and also by constructing cost-effectiveness acceptability curves (CEACS) to examine the probability that the intervention is cost-effective at different levels of willingness to pay (lambda). We additionally considered the expected value of perfect information (EVPI)—the expected value of eliminating uncertainty on all parameters—and the expected value of partial perfect information (EVPPI)—the expected value of eliminating uncertainty on individual parameters. Population EVPI and EVPPI were determined by estimating the number of patients who will benefit from the technology over its lifetime.

### Time horizon and discounting

Our base case model has a 28-day time horizon, informed by our clinical experts. The intervention is primarily a change to the inpatient environment and the relevant costs and outcomes relate to the period of skin graft ‘take’, so relative differences in costs and effects relating to the intervention are likely to occur in the short term, while the patient is hospitalised. This is in line with a previous model in a less severe burns population which had a 21-day time horizon justified on the basis of time to skin re-epithelisation and typical inpatient duration [10]. No discounting was applied to costs and benefits in the model due to the short time horizon being modelled. However, in the Population EVPI and EVPPI calculations over a 10-year lifetime horizon of the technology, an annual discount rate of 3.5% was applied [11].

#### Model structure

**Fig. 1:**
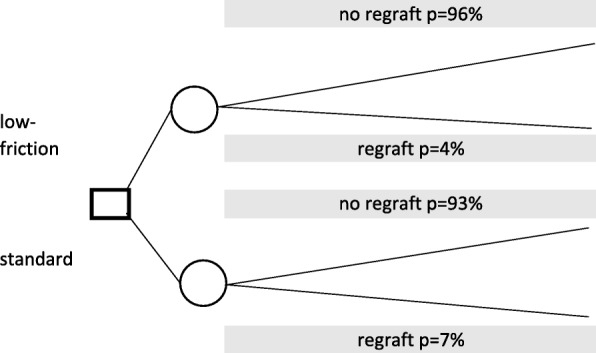
Model diagram. The decision node (square box) shows the option available to nurses: either to manage patients recovering from skin graft surgery on standard hospital bedding, or else place patients on low-friction ‘Silkie’ sheets. The probabilities of subsequent graft losses (%) emanate from the circular chance nodes. Expected payoffs (costs and QALYs) to the patient are in turn weighted by the probabilities

A simple probabilistic decision tree model was constructed with input from the clinical team at a tertiary burns service in Bristol, UK. In the specification of the model, patients are placed on either “Silkie” [low -friction] or standard bedding after skin graft surgery. Patients may then lose all or a sufficient proportion of their grafts such that they require a re-graft; otherwise, they were not re-grafted. Figure 1 describes the structure of the 28-day decision-tree which corresponds to the decision problem which could be addressed by a full RCT.

#### Model inputs

Data sources used to populate the model are described in the following sections. Input parameter values and distributions used in the probabilistic sensitivity analysis are presented in Table 1.

**Table 1:** Summary of inputs used in model

Variable	Estimates (credible interval)	Distribution	*n*	Source/assumption
Relative risk (regraft) [low-friction vs. standard]	0.574 (0.52, 0.63)	Lognormal (− 0.56,0.61)	221	Before and after comparison—Silkie feasibility study
P (regraft) low-friction	0.038 (0.013, 0.077)	Beta (4.96,125.04)	90	Prospective data collection—Silkie feasibility study
Intervention cost, £	115	Deterministic	131	Estimate based on unit costing approach
Cost (re-graft), £	19,321 (9092, 36,251)	Lognormal (9.81, 0.35)	131	“”
Cost (no regraft), £	9908 (8319, 11,705)	Lognormal (9.20, 0.09))	131	“”
Utility post surgery	0.353 (0.0056, 0.91)	Beta (0.77,1.41)	40	Prospective data collection—Silkie feasibility study
Utility no graft loss	0.508 (0.11, 0.90)	Beta (2.26, 2.19)	27	Prospective data collection—Silkie feasibility study
Utility graft loss	0.627 (0.57, 0.68)	Beta (179.95, 106.82)	2	
Discount rate	0.035 (−)	Deterministic	n/a	NICE methods of technology appraisal (2013)

### Relative risk of the low-friction environment (versus standard care)

Ideally, evidence on relative risks of re-graft would come from an RCT [12]. In the absence of this, comparative evidence from the Silkie before and after study was used. The need for re-grafting on low-friction bedding was a prospectively collected study outcome and the relative risk of low-friction versus standard was informed by the before and after comparison. Because the lack of concealed randomisation can lead to problems of comparability because of selection biases and potential confounders [13, 14] and because before/after designs are by definition prone to history bias [15], we performed multiple sensitivity analyses to assumptions on the magnitude and precision of the bias (see ‘Sensitivity analyses’).

### Absolute risk of graft loss associated with standard care

Absolute risk on standard sheets should come from representative contemporary practise. The Silkie study’s before element was informed by retrospective notes review of graft losses and this informed the absolute and therefore the relative risk used in the model (as described above).

### Cost of re-grafting

Ideally, the cost of re-grafting would come from micro-costing studies since such studies offer the most comprehensive and rigorous methods to capture the cost associated with a specific procedure [16]. In the absence of this, we sought to use national reference costs which provide a source of unit costs based on trust activity [17]. In our analysis of inpatient costs, we multiplied the cost of an NHS reference cost ‘inlier’ bed-day by length of stay to estimate inpatient care costs at the level of the patient. Bed-day costs were determined by application of the appropriate reference cost category for the procedure(s). In the absence of more detailed evidence, the cost of re-grafting was taken to be the incremental difference in the costs in patients who required re-grafting and those that did not, based on all patients (i.e. standard care and intervention arms) in the Silkie study.

### QALYs

Ideally, data on utilities would be routinely collected in the population with burn injury to allow for service evaluation and monitoring. A population-level sample would greatly increase the precision around the estimate of expected QALYs. However, measurement of health-related QoL is not routine in the UK burn populations, so the utility estimates for patients who did and did not undergo re-graft surgeries were identified from the perspective (intervention arm) of Silkie study. In the first year of the model, utilities were obtained from EQ-5D utility scores recorded at baseline and 28 days follow-up (the latter stratified according to re-graft status) in the intervention arm in study participants who consented to complete questionnaires.

### Analysis

The analysis was performed from the UK NHS healthcare perspective, and costs were reported in £ for the 2016 price year. We used a Monte Carlo simulation with 50,000 samples to propagate the joint uncertainty in model inputs into Net Benefit. In order to calculate the population level expected value of perfect information, we considered the technology relevance horizon for a technology such as this to be around 10 years. Given an estimated 1000 skin grafts per annum being carried out in England and Wales [2], expressing this in present value terms via application of a discount rate of 3.5% the expected discounted population over 10 years was 8608. We additionally calculated the EVPPI using generalised additive regression (GAM) and integrated nested Laplace approximation (INLA) using, due to higher computational demands of GAM and INLA, 10,000 of the Monte Carlo samples [18, 19].

### Sensitivity analyses

As the Silkie study used non-contemporaneous controls, it is likely the estimated relative risk and its confidence interval may be subject to bias; however, we do not know the extent of this bias. We explored the robustness of our conclusions to potential bias by presenting results for the mean bias (none, favour Silkie, favour standard), together with inflation or deflation of uncertainty (see Table 3). Details of distribution used for the relative risk of bias term are provided in Table 3.

### Model validation

The model was developed in R with the Bayesian Cost-Effectiveness Analysis package [20, 21]. Face validity of the model (and particularly the modelled assumptions) was established through discussion with the Silkie study management group and, health economic modellers. Internal validity was systematically checked by assessing the core calculations of the model and applying a series of logical checks to ensure that the direction of the model’s predictions were consistent. We additionally used Excel to validate our calculations with respect to Net Benefit and EVPI. External and cross validity was more difficult to establish, as there were no cost-utility or value of information studies in the target population with which to compare our results.

### Reporting

We used the CHEERS reporting guidelines for Economic Evaluation [22].

## Results

**Fig. 2:**
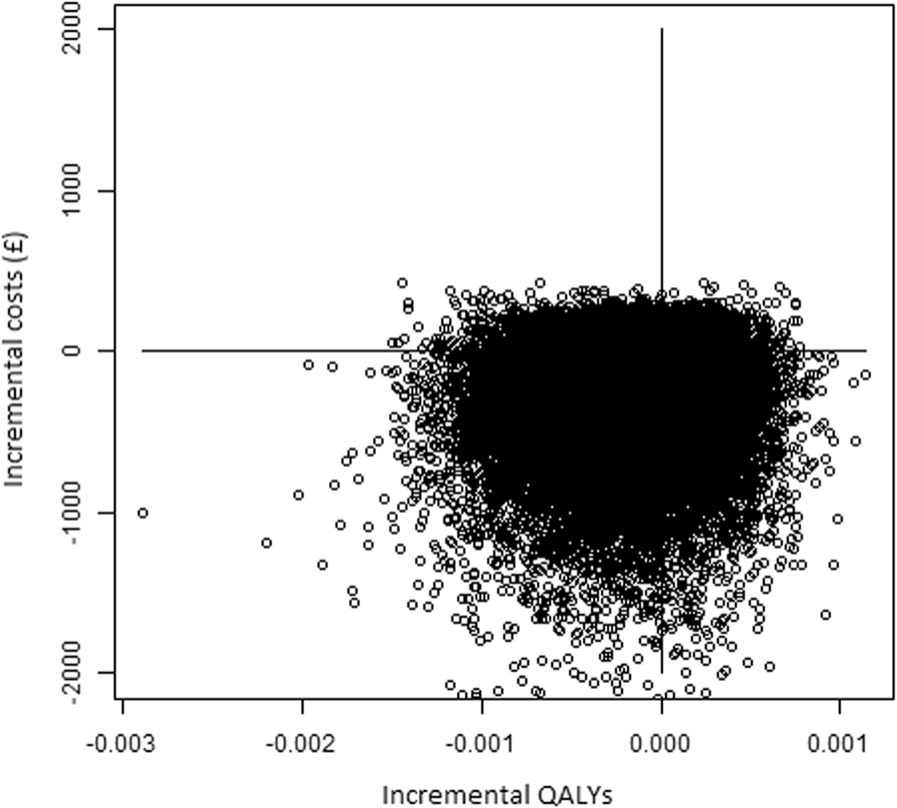
Scatterplot of cost and effect pairs for low-friction bedding compared to standard care (28 days)

**Fig. 3:**
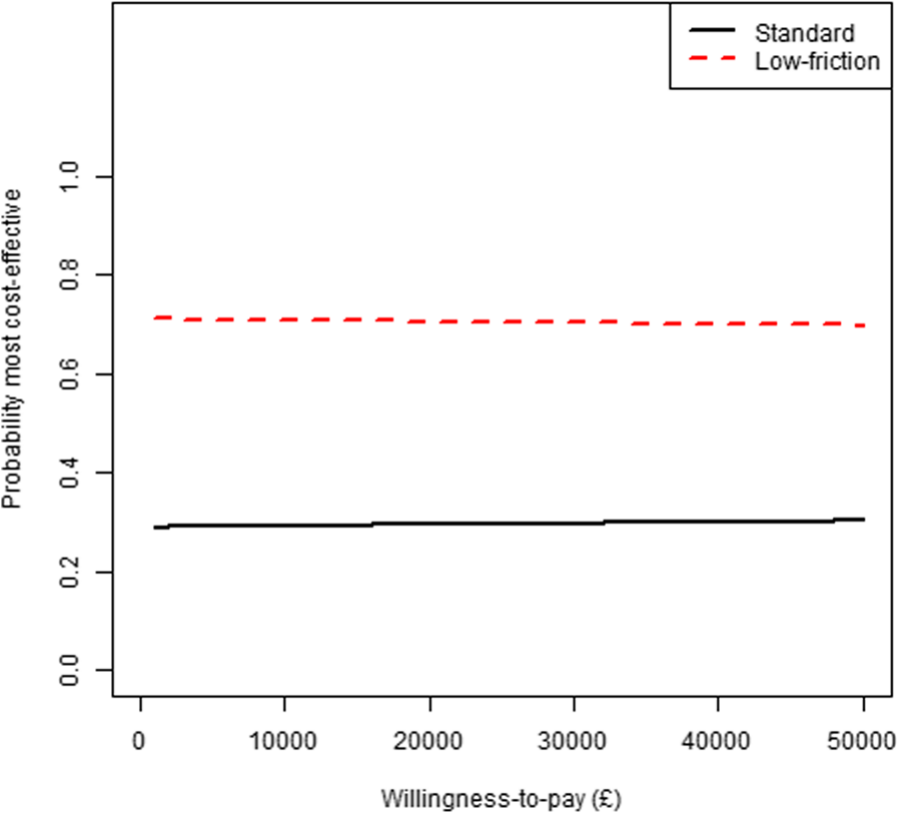
Cost-effectiveness acceptability curve

Table 2 presents the results from the probabilistic model over 28 days for the low-friction and standard bedding. The probabilistic analyses incorporate the uncertainty around the point estimates of input parameters (Fig. 2). In the base-case, where the Silkie study results are taken at face value, the expected Incremental Net Benefit was £151 in favour of low-friction bedding at a threshold of £20,000 per QALY gained. However, there was considerable uncertainty around this estimate (95%CrI £-142 to 814) suggesting that there is no clear optimal strategy based on ENB. Figure 3 presents the cost-effectiveness acceptability curves which shows that low-friction bedding has a 70.5% chance of being cost-effective at a threshold of £20,000 per QALY gained. Table 3 shows the Population EVPI over the 28-day time horizon is £20.29 per person at a threshold value of £20,000. For a decision relevance horizon of 10 years, this corresponds to an overall expected value of removing decision uncertainty for England and Wales of £174,675.

**Table 2:** Costs and outcomes of providing the low-friction environment and standard care (28 days)

Intervention	Standard	Low-friction	Incremental
Mean 28-day QALYs	0.033 (0.011, 0.060)	0.0332 (0.010, 0.060)	−0.00013 (−0.00073, 0.00034)
Mean 28-day costs (£)	10,536 (8786, 12,689)	10,382 (8748, 12,261)	− 154 (− 818, 139)
Expected net benefit at £ 20,000 per QALY	− 9870 (−12,072, − 8041)	− 9718 (−11,657, − 7999)	151 (−142, 814)

**Table 3:** Sensitivity analysis to bias in the relative risk (RR) for re-graft in low-friction relative to standard bedding

Scenario	Scenario parameters		Scenario results		Individual level value of information (£)					Population level value of information (£)	
	RR bias	RR	INB (£)	Probability low-friction cost-effective	EVPI	EVPPI bias	EVPPI RR	EVPPI Probability regraft	EVPPI cost regraft	EVPI population	EVPPI RR population
1. Face-value (base case)	1.00 (1.00, 1.00)	0.57 (0.52, 0.632)	151 (−142, 814)	0.71	20.29	NA	0.00	1.66	16.06	174,675	–
2. No bias, high uncertainty	1.00 (0.47, 1.88)	0.57 (0.27, 1.08)	237 (− 171, 1551)	0.65	30.69	12.61	13.45	0.57	13.85	264,165	115,789
3. Bias in favour low-friction, high uncertainty	0.80 (0.29, 1.76)	0.458 (0.17, 1.02)	490 (− 186, 2772)	0.75	25.02	7.96	9.96	0.06	12.16	215,354	85,755
4. Bias against low-friction, high uncertainty	1.20 (0.65, 2.03)	0.69 (0.37, 1.17)	94 (− 177, 919)	0.50	43.47	24.44	24.81	3.65	19.33	374,215	213,597
5. No bias, low uncertainty	1.00 (0.74, 1.31)	0.57 (0.42, 0.77)	166 (−141, 952)	0.69	21.74	0.62	0.82	1.41	15.51	187,103	7075
6. Bias in favour of low-friction, low uncertainty	0.80 (0.55, 1.13)	0.46 (0.31, 0.66)	334 (−156, 1581)	0.80	16.80	0.04	0.04	0.23	12.70	144,581	311
7. Bias against low-friction, low uncertainty	1.20 (0.94, 1.51)	0.69 (0.53, 0.88)	56 (− 131, 553)	0.51	32.64	6.01	7.27	7.05	22.81	280,986	62,608

*RR*: Relative risk of low-friction vs standard; *INB*: Incremental net benefit of low-friction vs standard; probability low-friction cost-effective is probability INB is greater than 0; *EVPI*: Expected value of perfect information; *EVPPI*: Expected value of partial perfect information. Final two columns extrapolate individual EVPI and EVPPI for RR to a total (discounted) population of 8608 patients over the 10-year lifetime for the technology

**Fig. 4:**
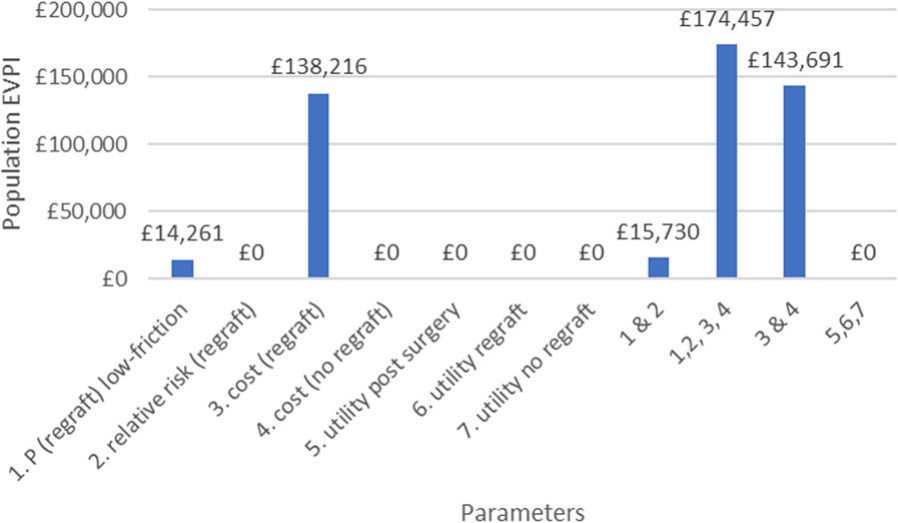
Expected value of perfect information in individual parameters

Figure 4 describes the EVPPI for individual parameters. This shows that the parameters causing most of the decision uncertainty are around costs and the absolute probability for re-grafting surgery, regardless of the sheeting environment. Table 3 also shows the results of the sensitivity analysis for bias in the relative risk, and implications in terms of expected net-benefit, the probability that low-friction is most cost-effective, EVPI and EVPPI parameters. EVPPI for further parameter sets under bias scenarios is provided in the Appendix. The probability that low-friction is most cost-effective is higher when bias is upwards (favouring low-friction) and when bias is least uncertain. We see that the EVPI is greatest when the benefit of low-friction vs standard is most uncertain (bias against low-friction bedding with high uncertainty, scenario 4) and lowest when the benefit is most certain (bias in favour of low-friction and low uncertainty, scenario 6). The parameters with largest EVPPI, and with EVPPI that changes most under bias scenarios, are the probability of re-graft, relative risk of re-graft, and the cost of re-graft; utilities appear to have little influence on decision making and are unaffected by assumptions about bias in the relative risk (see Appendix). The final column represents the value of an RCT exploring the relative risk, with no value in the base case but the highest value if we assume the observational evidence is biased against low-friction bedding and highly uncertain (scenario 4) or unbiased but highly uncertain (scenario 2). Regardless of the assumptions about bias, there does not seem to be value in an RCT of low-friction vs standard bedding. However, there may be value in conducting another type of study to collect re-graft costs as population EVPPI for this parameter was £138,216. This would be much cheaper and probably worthwhile (given the data would be relevant for other decision questions for such patients in the future).

## Discussion

This paper reports on the construction of a model to evaluate the cost-effectiveness of a low-friction environment in patients who have had recent skin graft surgery, and the potential value of future research. Our results suggest that there is no value in a future RCT regardless of how large we believe the bias from the before-after Silkie study to be because the cost of an RCT would far outweigh the benefits from it as measured by EVPPI. However, we cannot be sure which is the optimal treatment. This suggests that the consequences of using low-friction or standard bedding are not sufficiently high for us to be concerned if the wrong choice is made. However, we found that there may be value in studies collecting data on the cost of re-graft surgery and the absolute probability of re-graft (regardless of bedding type). These data could be used to update the model and revisit the decision on bedding, and also would be valuable to inform decisions on other interventions for this population in the future.

### Summary of findings elsewhere

We found no published evaluations in burn management using cost-effectiveness modelling. Brown, David et al. [23] published an economic evaluation of a distraction-based intervention ‘Ditto’ in paediatric burn care in Brisbane, Australia based on a small trial (*n* = 75). The study reported a 95% probability that it was cost-effective compared with standard care, but the results are not easily comparable since they report cost (AU$) per 1-day reduction in re-epithelialization. Elsewhere, Tuffaha et al. identify the EVPI for the decision to adopt negative pressure wound therapy at AUD 2.7 million, which places a much greater value on more research than for our study [24]. We found evidence of an unpublished UK economic evaluation for a spray-on skin system in patients with burn injury [10] but the model developed was only applicable to patients with partial burns that do not require re-grafting due to a lack of evidence in grafted patients and did not consider value of information. Additionally, we are aware of modest literature on the costs associated with burn injury in child and adult populations. However, these studies are typically either very small or report costs based on different healthcare systems so may not be generalisable [25–30].

## Limitations

One limitation is that we present modelled results drawing on a retrospective comparison of patients nursed on standard bedding, in a preceding 12-month period in the same hospitals. This lack of a contemporaneous and prospectively collected comparison group is a key limitation of inference on the basis of feasibility study results. While we explored these in scenario analyses the nature of bias (some which may be unobservable) is that we do not know if the analyses we conducted are reasonable and explored the full range of possibilities.

We note that the short-time horizon of this model made it unlikely that we would identify differences in QALYs, and this may mean that we did not consider the inclusion of potentially relevant effects. These effects could be substantial if repeat skin grafts cause long-term or permanent scarring and the utility decrement (and the associated loss in QALYs) can be quantified. Further work could set out to collect data on utilities or elicit informative priors to describe uncertainty in these parameters [31] in order to populate a long-term model. Any future model development should foreseeably involve patients and the public to validate such long-term assumptions.

## Conclusion

Our model for the UK population suggests that a further definitive trial is not worthwhile and instead low-cost research audits should focus on the re-graft rates and cost of graft surgeries.

## Data Availability

The R code for the model is available from the corresponding author on reasonable request.

## Appendix

**Table 4:** Individual and group EVPPI under all bias scenarios

	Probability regraft	Relative risk	Cost regraft	Cost no regraft	Utility post surgery	Utility following regraft	Utility following no regraft	Probability regraft and relative risk	Probability regraft, relative risk, and all costs.	All costs	All utilities	Total
1. Face value (base case)	1.66	0.00	16.06	0.00	0.00	0.00	0.00	1.83	20.27	16.69	0.00	20.29
2. No bias, high uncertainty	0.57	13.45	13.85	0.00	0.00	0.00	0.00	15.83	31.11	14.25	0.00	30.69
3. Bias in favour low-friction, high uncertainty	0.06	9.96	12.16	0.00	0.00	0.00	0.00	10.94	26.35	13.20	0.00	25.02
4. Bias against low-friction, high uncertainty	3.65	24.81	19.33	0.00	0.00	0.00	0.00	28.69	43.49	19.65	0.00	43.47
5. No bias, low uncertainty	1.41	0.82	15.51	0.00	0.00	0.00	0.00	3.77	21.71	16.17	0.00	21.74
6. Bias in favour of low-friction, low uncertainty	0.23	0.04	12.70	0.00	0.00	0.00	0.00	0.88	16.76	13.64	0.00	16.80
7. Bias against low-friction, low uncertainty	7.05	7.27	22.81	0.01	0.00	0.00	0.00	14.30	32.63	23.25	0.00	32.64

Probability re-graft is the (absolute) probability of re-graft (as determined by the proportion of patients with skin graft failures on low-friction bedding). Relative risk is the ratio of the risks of regraft due to skin graft failure (low-friction vs standard bedding)

## Abbreviations

CrI: Credible interval
ENB: Expected net benefit
EVPI: Expected value of perfect information
EVPPI: Expected value of perfect partial information
iBID: National Burns Injury Database
NHS: National Health Service
QALY: Quality-adjusted life year
RCT: Randomised controlled trial
